# 91726 Screening for Obesity related renal damage in adolescent women - Body Surface area matters

**DOI:** 10.1017/cts.2021.470

**Published:** 2021-03-30

**Authors:** D. Bielopolski, N. Singh, O.S. Bentur, Y. Renert-Yuval, R. MacArthur, K. Vasquez, D.S. Moftah, R.D. Vaughan, R.G. Kost, J.N. Tobin

**Affiliations:** 1 The Rockefeller University Center for Clinical and Translational Science, New York, NY; 2 Clinical Directors Network (CDN), New York, NY

## Abstract

ABSTRACT IMPACT: This change will improve primary care physicians and pediatrics ability to identify, intervene and prevent obesity related renal damage in the vulnerable population of young adults OBJECTIVES/GOALS: Obesity related glomerulopathy has a reversible stage manifested as hyperfiltration. Early intervention depends on the ability to identify hyperfiltration. Hyperfiltration prevalence is underestimated using the currently recommended formula We investigated whether calculating BSA-adjusted GFR will more readily identify hyperfiltration. METHODS/STUDY POPULATION: We extracted data from a large urban, multi-institutional Electronic Health Records (EHR) clinical data research network to construct an EHR data base of 60,549 women and girls ages 12-21 years from the New York metropolitan area. EGFR was calculated in two ways, 1) according to age appropriate formula, and 2) according to age appropriate formula and adjusted to body surface area (BSA). BMI-for-age values were classified according to the World Health Organization schema and grouped according to the CDC definitions. BSA was calculated according to the Du-Bois formula. Hyperfiltration was defined by a threshold of 135ml/min. The Bland Altman method assessed the agreement between formulas across the different BMI groups. RESULTS/ANTICIPATED RESULTS: Serum creatinine values were similar across different BMI groups. Comparing eGFR values, hyperfiltration rates were similar across BMI groups, ranging between 4%-6.6%. BSA-adjusted GFR was different across BMI groups: hyperfiltration rates were 0.81% for the underweight group, 2.56% for the normal weight, 12.18% for the overweight and 39% in the obese group. This trend of hyperfiltration paralleled the the rise in urine creatinine across BMI groups. DISCUSSION/SIGNIFICANCE OF FINDINGS: BSA-adjusted GFR more sensitively detects hyperfiltration due to obesity than does eGFR. Calculating BSA-adjusted GFR will improve primary care and pediatric physicians’ ability to identify, intervene and prevent early ORG. Changes in body composition may account for the increasing discordance between BSA-adjusted and eGFR as BMI rises.

